# Redo axillary artery cannulation in aortic reoperations: Technical variations and implications for optimal outcomes

**DOI:** 10.1016/j.xjtc.2025.05.017

**Published:** 2025-06-05

**Authors:** Suguru Ohira, Gabrielle Amar, Sooyun Caroline Tavolacci, Masashi Kai, Ramin Malekan, Junichi Shimamura, Steven L. Lansman, David Spielvogel

**Affiliations:** a Division of Cardiothoracic Surgery, Department of Surgery, Westchester Medical Center, New York Medical College, Valhalla, NY; b Department of Cardiothoracic Surgery, New York Medical College, Valhalla, NY; c Department of Cardiac Surgery, Hartford Hospital, Hartford, Conn; d Division of Cardiac Surgery, Boston Deaconess Medical Center, Boston, Mass

**Keywords:** aorta, reoperation, axillary artery

## Abstract

**Objectives:**

Recannulation of the right axillary artery (Redo-AX) is a valuable yet underutilized technique in aortic reoperations. The present study sought to analyze the outcomes of 1 of the largest redo AX cannulations experiences.

**Methods:**

From February 2005 to December 2024, AX cannulation was planned for 804 aortic repairs and analyzed according to the intention-to-treat principle. Fifty patients had Redo-AX, whereas 754 patients had primary AX cannulation. Cannulation-related events included technical failure, vascular injury, additional vascular rep, and iatrogenic retrograde dissection.

**Results:**

This cohort included 196 redo sternotomies (24.4%) and 381 type A aortic dissections (47.4%). Among the 50 Redo-AX procedures, 46 patients had direct AX cannulation, and 4 patients had the side-graft technique in their initial surgery. Forty-five patients were successfully cannulated for cardiopulmonary bypass. Two patients underwent the side-graft technique with a graft extension, and direct AX cannulation was performed in 43 patients via arteriotomy (n = 40), the Seldinger technique (n = 2), and direct cannulation through an old polyethylene terephthalate graft (n = 1). The overall rate of cannulation-related events was 2.1% (17 out of 804), and the rate of cannulation site shift was 2.7% (22 out of 804). Cannulation-related events (10% vs 1.6%; *P* < .001) were significantly more common in the Redo-AX group. Operative mortality was comparable between groups (Redo-AX, 0% vs Primary-AX, 4.8%; *P* = .220), as was the incidence of stroke (0% vs 4.9%, *P* = .209).

**Conclusions:**

Redo-AX is a durable approach for complex redo aortic cases. Careful preoperative evaluation and certain surgical expertise are paramount to achieving optimal outcomes.

## Graphical abstract

**Figure:**
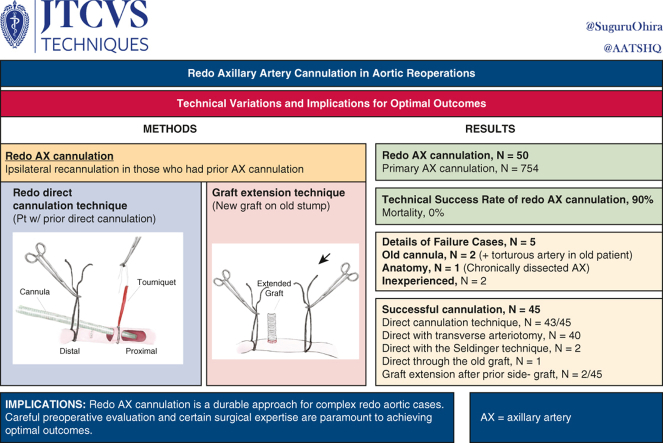

**Figure:**
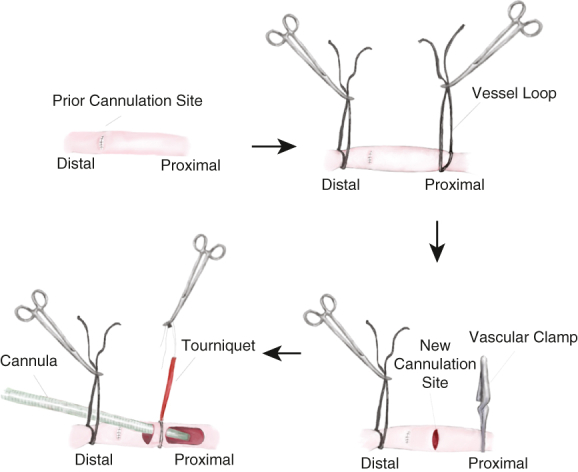
Redo direct cannulation through a transverse arteriotomy.

**Central Message**

Redo cannulation of the axillary artery in patients with a history of axillary artery cannulation is a durable and effective approach for complex redo aortic cases.

**Perspective**

Recannulation of the ipsilateral AX is an effective strategy for establishing cardiopulmonary bypass in redo aortic cases, although this technique requires a certain level of surgical expertise. Both the direct and side-graft techniques for AX cannulation complement each other, allowing for individualized approaches based on the patient's pathology and anatomy.

The number of thoracic aortic reoperations continues to grow due to improvements in initial surgical outcomes and the fact that patients undergoing complex thoracic aortic repair may require lifetime management.^1^, ^2^, ^3^, ^4^ Preparation for peripheral cannulation is essential for safely performing these complex reoperations because reentry of the sternum and dissection around great vessels can be challenging.^1^, ^2^, ^3^, ^4^, ^5^, ^6^ Cannulation of the axillary artery (AX) is often utilized in aortic repairs and is performed using 2 main techniques: direct cannulation or the side-graft technique.^7^, ^8^, ^9^, ^10^, ^11^ The potential benefits of right AX cannulation include arterial outflow through a disease-free artery, antegrade flow through the aortic arch, and the ability to transition smoothly to antegrade cerebral perfusion (ACP) if necessary.^3^^,^^7^, ^8^, ^9^, ^10^ The femoral artery remains the most common cannulation site; however, certain patients are unsuitable due to diseased iliofemoral vessels, significant atherosclerosis in the thoracic or abdominal aorta, or a history of multiple femoral accesses.^5^^,^^6^ Recannulation of the right AX (Redo-AX) involves recannulating the ipsilateral AX in patients with a prior AX cannulation, making it a viable option for those requiring reoperative sternotomy.^12^, ^13^, ^14^, ^15^ This approach allows controlled chest reentry while maintaining the option for ACP in case deep hypothermic circulatory arrest is needed for a challenging reentry. To date, there is a paucity of data regarding Redo-AX in patients who previously underwent AX cannulation. This study aims to analyze our outcomes of Redo-AX, a technique that may be underutilized despite its usefulness.

## Patients and Method

This study is a retrospective analysis and was approved by the New York Medical College Internal Review Board, with a waiver of individual consent (No. 14209; approval date May 2, 2020). From February 2005 to December 2024, AX cannulation was planned for 804 aortic repairs according to the intention-to-treat principle. Fifty patients with a history of prior right AX cannulation underwent recannulation of the ipsilateral artery since July 2011.

### Surgical Techniques

Our direct cannulation technique was previously reported.^10^ An infraclavicular incision was made along the clavicle to access the second portion of the AX. In Redo-AX, an infraclavicular incision was made precisely along the old incision line to access the existing scar.^8^^,^^10^ The AX was then exposed and cannulated as described below.

### Redo-AX in Patients With Prior Direct AX Cannulation

#### Redo direct cannulation through a transverse arteriotomy

**Figure 1:**
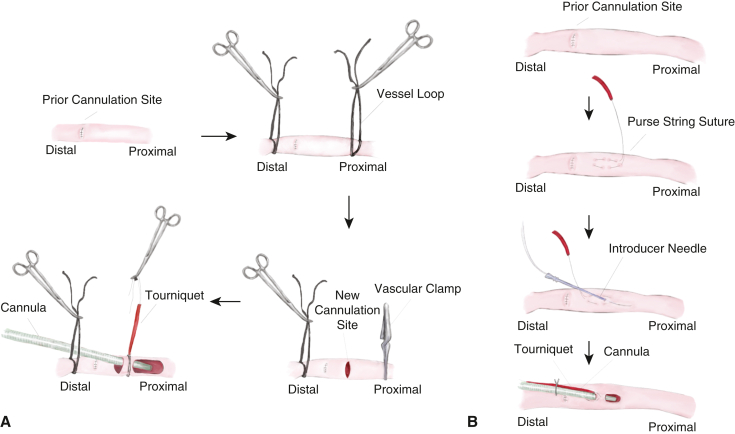
Variations of redo cannulation techniques in patients with prior history of direct axillary artery (AX) cannulation. A, After proximal and distal control with vessel loops, the proximal portion of the AX was occluded with a vascular clamp. A transverse arteriotomy was made, and an arterial cannula was inserted. The proximal vessel loop with a *red* rubber tourniquet was snared and secured. B, Alternatively, the Seldinger technique can be used if the AX is not *small*. Longitudinal purse string is placed, and the same straight cannula is used. This technique is useful in cases of dense adhesion to minimize dissection around the artery.

After proximal and distal control with vessel loops, the proximal segment of the artery was occluded with a vascular clamp (Video 1 and Figure 1, A. ). A transverse arteriotomy was made either proximally or distally to the previous cannulation site. A straight arterial cannula (OptiSite cannula 18, 20, or 22 Fr; Edwards Lifesciences) was inserted under direct vision while gradually releasing the vascular clamp. The proximal vessel loop, secured with a red rubber tourniquet, was snared and tied to the arterial cannula. For decannulation, the artery was primarily repaired using running 6-0 polypropylene suture.

#### The open Seldinger technique

A diamond-shape purse string was placed on the AX. The same straight cannula was advanced over the guidewire following serial dilatations. This technique may be particularly useful in cases of dense adhesion to minimize dissection around the artery (Figure 1, *B*). After decannulation, the purse-sting suture was tied, followed by a figure-of-8 suture.

#### The side-graft technique

The standard side-graft technique can be performed; however, we have not performed this technique in patients with prior history of direct AX cannulation.

### Prior AX Cannulation Using Side-graft Technique

After proximal and distal control, the AX was clamped. The distal end of the old graft was excised, and organized thrombi were carefully removed. Irrigation of the inside of the old graft is crucial to prevent embolization.

#### Extension of a polyethylene terephthalate graft

**Figure 2:**
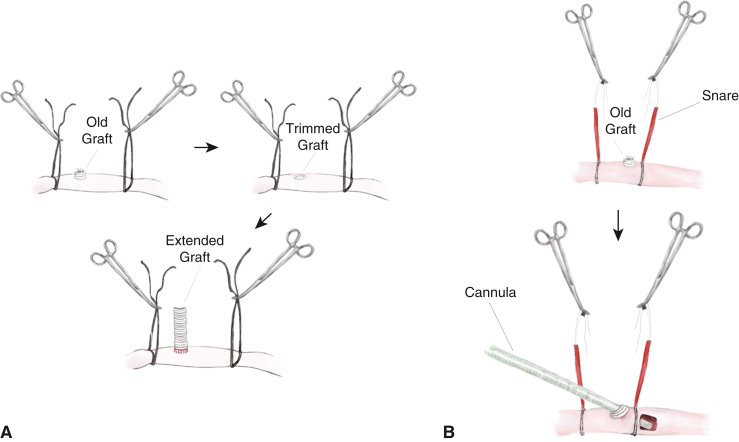
Examples of redo axillary artery (AX) cannulation techniques in patients with a prior history of the side-graft technique. After proximal and distal control, clots inside the old graft were removed. A, The old graft was trimmed as short as possible. A new graft was anastomosed to incorporate the arterial wall, old polyethylene terephthalate graft, and new graft. B, Alternatively, a straight arterial cannula can be inserted through the old graft, like the direct cannulation technique. After decannulation, the stump of the graft is oversewn.

The old polyethylene terephthalate (PTE) graft was trimmed, leaving 2 to 3 crimps intact. A new PTE graft (8 or 10 mm) was anastomosed to the arteriotomy and the remaining portion of the old graft, incorporating the arterial wall, old graft, and new PTE graft using 4-0 or 5-0 polypropylene sutures (Figure 2, *A*). The other end of the PTE graft was connected to the arterial tubing. For decannulation, the side-graft was ligated using silk ties or closed with polypropylene sutures.

#### Direct cannulation through the old graft

After proximal and distal control using vessel loops, a straight arterial cannula was inserted through the graft and advanced into the AX (Figure 2, *B*). Both proximal and distal AX segments were snared similar to the direct AX cannulation technique described earlier. After decannulation, the distal end of the graft was oversewn using a running polypropylene suture.

#### Direct cannulation distal to the old anastomosis

A direct cannulation technique could also be performed distal to the previous PTE graft anastomosis, provided sufficient length of the AX is exposed.

We do not routinely initiate cardiopulmonary bypass before reentry unless the patient has a pseudoaneurysm in close proximity to the sternum. In such cases, cardiopulmonary bypass is established before reentry, followed by the insertion of a left ventricular vent through the apex via small left thoracotomy.

### End Points and Definitions

The primary outcome was intraoperative AX cannulation-related events, including technical failure of arterial cannulation, vascular injury, vascular repair requiring more than primary closure, and iatrogenic retrograde dissection from AX cannulation. Postoperative AX cannulation-related complications included brachial plexus symptoms, ipsilateral arm ischemia, hyperperfusion (ie, reperfusion injury after direct cannulation, and/or hyperperfusion after side branch cannulation), and AX wound complications.

Secondary outcomes included hospital mortality and stroke, defined as new brain injury evident either clinically or radiographically. During the postoperative period, vascular Doppler ultrasound was routinely performed on each arm and leg in addition to standard physical examinations.

### Statistical Analysis

Continuous variables are presented as median (first; third quartile) and were compared using the Mann-Whitney *U* test. Categorical variables were expressed in percentages and are compared using the χ^2^ or Fisher exact test (n < 5). Statistical analyses were performed using SPSS 24.0 (IBM-SPSS Inc).

## Results

### Characteristics and Early Outcomes

The median age was lower in the Redo-AX group (60.5 years; range, 49.8-66.3 years vs 65 years; range, 54-74 years; *P* = .020, with a higher prevalence of connective tissue disorders (16% vs 2.1%; *P* < .001) (Table 1). All patients in the Redo-AX group underwent redo sternotomy, whereas 146 patients (19.4%) in the Primary-AX group had prior sternotomy. There were no type A dissection patients in the Redo-AX group. Aortic valve insufficiency was more common in the Primary-AX group (18% vs 42.7%; *P* < .001). The majority of patients (n = 796 [99.0%]) received direct AX cannulation in this cohort.

**Table 1:** Demographic characteristics of patients

Variables	All (N = 804)	Redo AX (n = 50)	Primary AX (n = 754)	*P* value
Age (y)	64 (54-74)	60.5 (49.8-66.3)	65 (54-74)	.020
Male	545 (67.8)	35 (70)	510 (67.6)	.729
Body surface area (m^2^)	2.00 (1.82; 2.20)	1.95 (1.78; 2.19)	2.00 (1.82; 2.20)	.985
Body mass index	27.7 (24.5-31.9)	27.1 (23.9-30.4)	27.7 (24.5-31.9)	.233
Connective tissue disease	24 (3.5)	8 (16)	16 (2.1)	<.001
Smoking history	168 (20.9)	3 (6)	165 (21.9)	.013
Coronary artery disease	168 (20.9)	10 (20)	158 (21.0)	.872
Hypertension	652 (81.1)	45 (90)	607 (80.5)	.140
Diabetes mellitus	105 (13.1)	5 (10)	100 (13.3)	.655
Atrial fibrillation	90 (11.2)	7 (14)	83 (11.0)	.516
Peripheral vascular disease	26 (3.2)	1 (2)	25 (3.3)	.923
Cerebrovascular disease	55 (6.8)	7 (14)	48 (6.4)	.038
Chronic kidney disease	71 (8.8)	8 (16.0)	63 (8.4)	.065
Ejection fraction <50%	78 (9.7)	4 (8)	74 (9.8)	.863
Bicuspid aortic valve	62 (12.0)	2 (10)	60 (12.1)	1.00
Aortic stenosis	80 (10.0)	4 (8.0)	76 (10.1)	.634
Aortic insufficiency	331 (41.2)	9 (18)	322 (42.7)	<.001
Type A aortic dissection	381 (47.4)	0	381 (50.5)	<.001
Redo sternotomy	196 (24.4)	50 (100)	146 (19.4)	<.001
Cannulation type				.008
Direct with angled cannula	560 (69.7)	27 (54)	533 (70.7)	
Direct with straight cannula	236 (29.4)	21 (42)	215 (28.5)	
Polyethylene terephthalate side-graft	8 (1.0)	2 (4)	6 (0.8)	

Values are presented as n (%), median (range), or median (25%; 75% quartile).

*AX*, Axillary artery.

### Operative Outcomes

The details of aortic repairs were comparable between the 2 groups (Table 2) except that the Redo-AX group underwent more extensive arch repairs, including partial or total arch repair (Redo-0AX, 76% vs Primary-AX, 20.4%; *P* < .001). Aortic root replacement was performed in approximately one-third of patients. The cardiopulmonary bypass time was longer in the Redo-AX group (222 minutes; range, 201.8-249 minutes vs 173 minutes; range, 138-221 minutes; *P* < .001), whereas myocardial ischemic time was comparable between groups. Selective ACP was utilized more frequently in the Redo-AX group (84% vs 35.1%; *P* < .001).

**Table 2:** Operative outcomes

Variables	All (N = 804)<	Redo AX (n = 50)	Primary AX (n = 754)	*P* value
Any arch repair	706 (87.8)	44 (88)	662 (87.8)	.966
Total or partial arch repair	192 (23.9)	38 (76)	154 (20.4)	<.001
Aortic root replacement				.32
Bentall procedure	261 (32.5)	12 (24)	249 (33)	–
Valve-sparing root replacement	18 (2.2)	2 (4)	16 (2.1)	–
Coronary artery bypass grafting	141 (17.5)	5 (10)	136 (18.0)	.209
Aortic valve replacement	54 (2.0)	1 (2)	53 (7.0)	.278
Other valve procedure	35 (4.4)	5 (10)	30 (4.0)	.096
Selective antegrade cerebral perfusion	307 (38.2)	42 (84)	265 (35.1)	<.001
Cardiopulmonary bypass time (min)	178 (141; 224)	222 (201.8; 249)	173 (138; 221)	<.001
Myocardial ischemic time (min)	101 (66.3; 139.8)	102 (85; 136.3)	101 (65.8; 140)	.316
Cannulation site shift	22 (2.7)	5 (10)	17 (2.3)	.005
Cannulation-related events	17 (2.1)	5 (10)	12 (1.6)	<.001
Technical failure∗	11 (1.4)	4 (8)	7 (0.9)	<.001
Vascular injury∗	9 (1.1)	2 (4)	7 (0.9)	.192
Retrograde dissection	1 (0.1)	0	1 (0.1)	1.00
Mortality	36 (4.5)	0	36 (4.8)	.220
Stroke	37 (4.6)	0	37 (4.9)	.209
Reexploration for bleeding	33 (4.1)	4 (8)	29 (3.8)	.287
Respiratory complication	114 (14.2)	10 (20)	104 (13.8)	.223
Renal failure	43 (5.3)	2 (4)	41 (5.4)	1.00
Atrial fibrillation	90 (17.5)	4 (20)	86 (17.4)	1.00
Brachial plexus symptom	7 (0.9)	1 (2)	6 (0.8)	.919
Axillary wound complication	5 (0.5)	1 (2)	4 (0.5)	.725
Ipsilateral arm ischemia	0	0	0	1.00
Arm hyperperfusion	0	0	0	1.00

Values are presented as n (%), or median (25%; 75% quartile).

*AX*, Axillary artery.

∗ Some patients overlapped each other.

Cannulation site shifts and cannulation-related events, including technical failures, were more frequent in the Redo-AX group (10% vs 1.6%; *P* < .001). The overall incidence of vascular injury was 1.1% (n = 9). One retrograde dissection occurred in the Primary-AX group (0.1%), whereas no patient in the Redo-AX group experienced retrograde dissection. Operative mortality was comparable between groups (Redo-AX, 0% vs Primary-AX, 4.8%; *P* = .220), as was the incidence of stroke (0% vs 4.9%; *P* = .209). The incidence of postoperative cannulation-related complications was low, including brachial plexus symptoms and axillary wound complications. No cases of ipsilateral arm ischemia were observed.

### Outcomes of Redo-AX

The median interval from the prior operation to redo surgery was 2.6 years (range, 0.8-6.3 years) (Table 3). Four patients (n = 4 [8%]) had previous AX cannulation performed at outside hospitals using the side-graft technique. Third-time Redo AX was performed in 4 patients (8%). One patient experienced vascular injury during exposure of the AX, resulting in 49 patients being successfully cannulated. The success rate of right AX recannulation was 90% (n = 45 out of 50). Of these 45 patients, the direct cannulation technique was performed in 43 patients, including the following techniques: standard technique with a transverse arteriotomy, n = 40; the Seldinger technique, n = 2; and direct cannulation through the old PET graft, n = 1. The side-graft technique was used in 2 patients with a history of prior AX cannulation using a PET graft.

**Table 3:** Technical details of redo axillary artery (AX) cannulation (N = 50)

Detail	Result
Interval from prior AX cannulation (y)	2.6 (0.8; 6.3)
Prior surgery at outside hospital	4 (8)
Prior direct AX cannulation	46 (92)
Prior AX cannulation with the side-graft	4 (8)
Third time redo AX cannulation	4 (8)
Redo AX cannulation planned	50 (100)
AX cannulated	49 (97.5)
Direct cannulation technique attempted	47 (94)
Angled arterial cannula	26/47
Straight arterial cannula	21/47
Any cannulation site shift from the right AX	5/50 (10)
During exposure	1
During cannulation	3
After cannulation	1
Successful redo right AX cannulation	45/50 (90)
Direct right AX cannulation	43/45
With a transverse arteriotomy	40
With the Seldinger technique	2
Through the old polyethylene terephthalate graft	1
Side-graft technique	2/45

Values are presented as n (%) or median (25%; 75% quartile).

**Figure 3:**
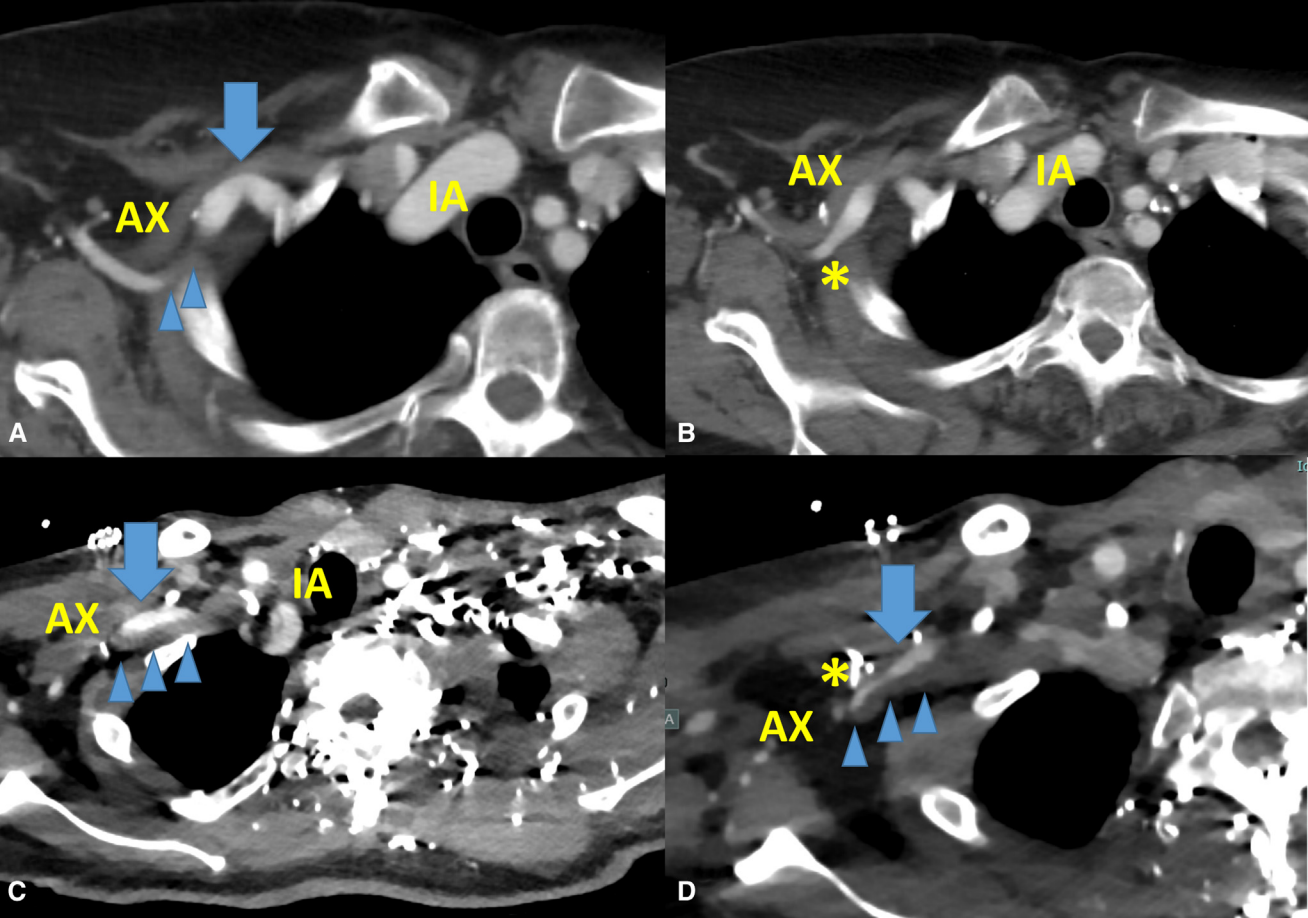
Failure cases of redo axillary artery (AX) cannulation. A and B, An 83-year-old woman with chronic dissection. A torturous AX to the subclavian artery was noted. The asterisk showed the old cannulation site. Redo direct axillary cannulation with an angled cannula was unsuccessful. The presumable cause was the acute turn of a proximal AX (*arrow*), which does not allow insertion of an arterial cannula. C, and D, A 45-year-old man with prior history of type A dissection repair. Preoperative computed tomography angiography showed a dissected AX and right subclavian artery with a thrombosed false lumen (*arrowheads*). A true lumen (*arrow*) of the right AX was 4.5 mm in diameter. Redo direct AX cannulation was performed but arterial back flow was insufficient. *IA*, Innominate artery. ∗The old cannulation site.

All failure cases (n = 5) had prior direct AX cannulation (Table 4). Redo direct AX cannulation was initially planned for all failure cases. Three failures (patients 1 through 3) occurred during arterial cannula insertion where an angled cannula was used. One of these 3 patients had a torturous AX (Figure 3, *A*). One patient (patient 4) had a dissected AX and subclavian artery with a thrombosed false lumen. This patient was initially cannulated using the direct cannulation technique, but the cannulation site was switched due to poor arterial backflow (Figure 3, *B*). One patient (patient 5) developed a vascular injury while dissecting out the side wall of AX, necessitating a switch in cannulation site to the innominate artery. All failure cases had a normal AX diameter (>7.5 mm), which is approximately equal to a 23 Fr cannula.

**Table 4:** Details of failure or conversion cases of redo axillary artery (AX) cannulation

Patient	Redo operation	Redo cannulation technique	When aborted	Event	Presumable reasons	AX diameter (mm)	Cannulation site as a result	Outcome
1:66 F	TAR ET	Direct arteriotomy (Angled)	During cannulation	Injury	Less experience Use of an angled cannula	7.7	Aortic arch	Discharge No stroke
2:83 F	TAR ET TVR	Direct arteriotomy (Angled)	During cannulation	Could not advance cannula	Torturous AX and SCA Use of an angled cannula	9.5	Right CCA	Discharge No stroke
3:43 F	Bentall TAR ET	Direct arteriotomy (Angled)	During cannulation	Could not advance cannula	Use of an angled cannula	10.7	IA	Discharge No stroke
4:45 M	TAR ET	Direct arteriotomy (Straight)	After cannulation	Poor back flow	Chronic dissection of AX and SCA	9.5 (TL 4.2)	LAX	Discharge No stroke
5:65 M	Bentall Hemiarch	–	Taping AX	Injury of artery	Less experience	9.8	IA	Discharge No stroke

*F*, Female; *TAR*, total arch repair; *ET*, elephant trunk; *TVR*, tricuspid replacement; *SCA*, subclavian artery; *CCA*, common carotid artery; *IA*, innominate artery; *M*, male; *TL*, true lumen; *LAX*, left axillary artery.

## Discussion

### Options for Redo-AX

In this study, we reviewed 1 of the largest series of Redo-AX procedures for aortic reoperation. The University of Freiburg group previously reported their experience with 32 Redo-AX procedures, demonstrating excellent outcomes.^12^ They primarily used a new PET graft anastomosed to the old graft stump, into which an arterial cannula was inserted.^12^ The Duke group reported a similar approach, incorporating both recannulation through the old graft and separate anastomosis of a new graft placed more distally.^13^ Although the side-graft technique is more commonly used for AX cannulation, our institution reported the safety and efficacy of direct AX cannulation in both elective and emergency cases.^10^ The potential benefits of the direct cannulation technique include expediency, hemostasis during cardiopulmonary bypass, and minimal dissection required around the artery.^3^^,^^9^^,^^10^ However, there may be concerns related to direct cannulation technique such as vascular injury or retrograde dissection, as previously reported by Sabik and colleagues.^8^ In the present study, the rates of retrograde dissection (0.1%) and vascular injury (1.1%) were favorable, despite our primary use of direct cannulation with a transverse arteriotomy in both primary and redo cannulation of the AX. The Seldinger technique is an alternative option that may be particularly useful in Redo-AX because it allows cannulation without requiring full proximal and distal control arterial control.^11^ However, the Seldinger technique can be challenging if the AX diameter is small because the purse-sting suture may cause narrowing of the artery after decannulation and also carries a risk of arterial injury or embolism related to wire manipulation.^11^ Therefore, direct recannulation technique with arteriotomy has been our preferred approach. Notably, the Seldinger technique was not available in the era of angled cannulas, which was another contributing factor. For patients with a history of prior direct AX cannulation, the side-graft technique may be a viable alternative.^8^^,^^9^ However, it is important to note that the side-graft technique is not without risks. The AX may be fragile or densely adherent, increasing the risk of intraoperative bleeding from the anastomosis or graft itself during cardiopulmonary bypass.^9^ One potential advantage of the side-graft technique over direct cannulation is that graft anastomosis can be performed before sternal reentry. However, this requires administration of heparin.^8^^,^^9^^,^^13^ In the case of a prior graft, we consider that extension of a graft is our first choice because direct recannulation of the old graft requires more extensive dissection around the artery. Instead of reopening the previous infraclavicular scar, surgeons may opt for a lateral approach for AX exposure. Possible techniques include an oblique or vertical incision along the lateral border of a pectoralis major muscle to expose the third portion of the AX lateral to the pectoralis minor muscle^16^; or axillary incision with ipsilateral arm abduction, as described by Ogino and colleagues.^17^ A potential drawback of these approaches is that the AX diameter may be smaller in these distal locations compared with the proximal artery.^16^^,^^17^ In redo aortic cases, femoral cannulation is also a widely used peripheral access approach.^5^^,^^6^ Several aortic centers report excellent outcomes of femoral artery cannulation.^5^^,^^6^ However, we prefer AX cannulation due to our extensive experience with this approach for aortic and cardiac cases, as well as for temporary mechanical circulatory support, including venoarterial extracorporeal membrane oxygenation, percutaneous microaxial pumps, and an intra-aortic balloon pumps.^18^ In addition, wound complications related to femoral cannulation are not negligible, especially in open femoral cannulation, and in patients with obesity, diabetes mellitus, or immunosuppression.^19^

### Implications From Failure Cases

Among the challenges of Redo-AX is the presence of dense adhesions around the artery, including the brachial plexus and axillary vein, which can lead to plexus symptoms or bleeding. In our series, the incidence of brachial plexus symptoms in the Redo-AX group was comparable to that in Primary-AX, and was similar to rates reported by the Freiburg group (3.1%) and the Duke group (2.6%). We encountered an AX injury while dissecting and taping the proximal part of the artery (case 5).

The failure cases were equally distributed throughout the study period. In 3 cases where technical failure occurred (cases 1-3), an angled cannula was used, but cannulation was aborted due to difficulties in insertion or advancement of the angled cannula. This issue arose because the wide, blunt tip of the angled cannula did not fit well within the small arteriotomy of the AX axillary of these patients. Insertion of a straight cannula with an inner sheath is less technically demanding for direct cannulation, even in acute type A aortic dissection cases, where the Seldinger technique is also an option. So far, we have not experienced any vascular injuries related to the inner sheath. Since August 2020, the angled arterial cannula was discontinued and is no longer commercially available. Since then, we have exclusively used straight arterial cannulas. Of note, the Seldinger technique was not our option until this straight cannula with inner sheath became available.

The failure of case 4 could have been prevented by selecting an alternative cannulation site. The patient had a dissection involving the innominate and right subclavian artery at the time of initial type A aortic dissection. Before the redo surgery, his preoperative computed tomography angiography scan showed that the right AX and proximal arteries were dissected with a thrombosed false lumen. Choosing a different cannulation site from the outset could have avoided the need for intraoperative cannulation site shifts. The 2 failure cases (cases 1 and 5) were performed by surgeons who were performing their first Redo-AX. Therefore, Redo-AX may require a certain level of experience, including proficiency in AX exposure, cannulation techniques, and reentry of redo cardiac or aortic cases.

Limitations of this study include its single-center, retrospective design with a small patient cohort and the absence of an ideal control group. Our center exclusively performs the direct insertion technique for AX cannulation. In most patients who had prior AX cannulations (92%), the initial operations were performed at our center. Therefore, the findings of this study might not be generalizable to other centers. Despite these limitations, we believe our study provides important insights.

## Conclusions

Our experience with the largest cohort of patients undergoing Redo-AX demonstrates that recannulation of the ipsilateral AX is an effective strategy for establishing cardiopulmonary bypass in redo aortic cases, although this technique requires a certain level of surgical expertise. The liberal use of various Redo-AX techniques, along with of alternative arterial cannulation site options, is paramount for successful outcomes in complex cases. Both the direct and side-graft techniques for AX cannulation complement each other, allowing for individualized approaches based on the patient's pathology and anatomy.

## Conflict of Interest Statement

The authors reported no conflicts of interest.

The *Journal* policy requires editors and reviewers to disclose conflicts of interest and to decline handling and reviewing manuscripts for which they may have a conflict of interest. The editors and reviewers of this article have no conflicts of interest.

## Supplemantary Data

Video 1 Redo right axillary artery cannulation using direct cannulation technique in a patient with prior direct axillary cannulation. Video available at: https://www.jtcvs.org/article/S2666-2507(25)00237-8/fulltext.
