# Supplementary figures and images for: Redo axillary artery cannulation in aortic reoperations: Technical variations and implications for optimal outcomes
Source: JTCVS Tech. 2025 Jun 5;32:1–9. doi: 10.1016/j.xjtc.2025.05.017 (PMC12347680; doi:10.1016/j.xjtc.2025.05.017)

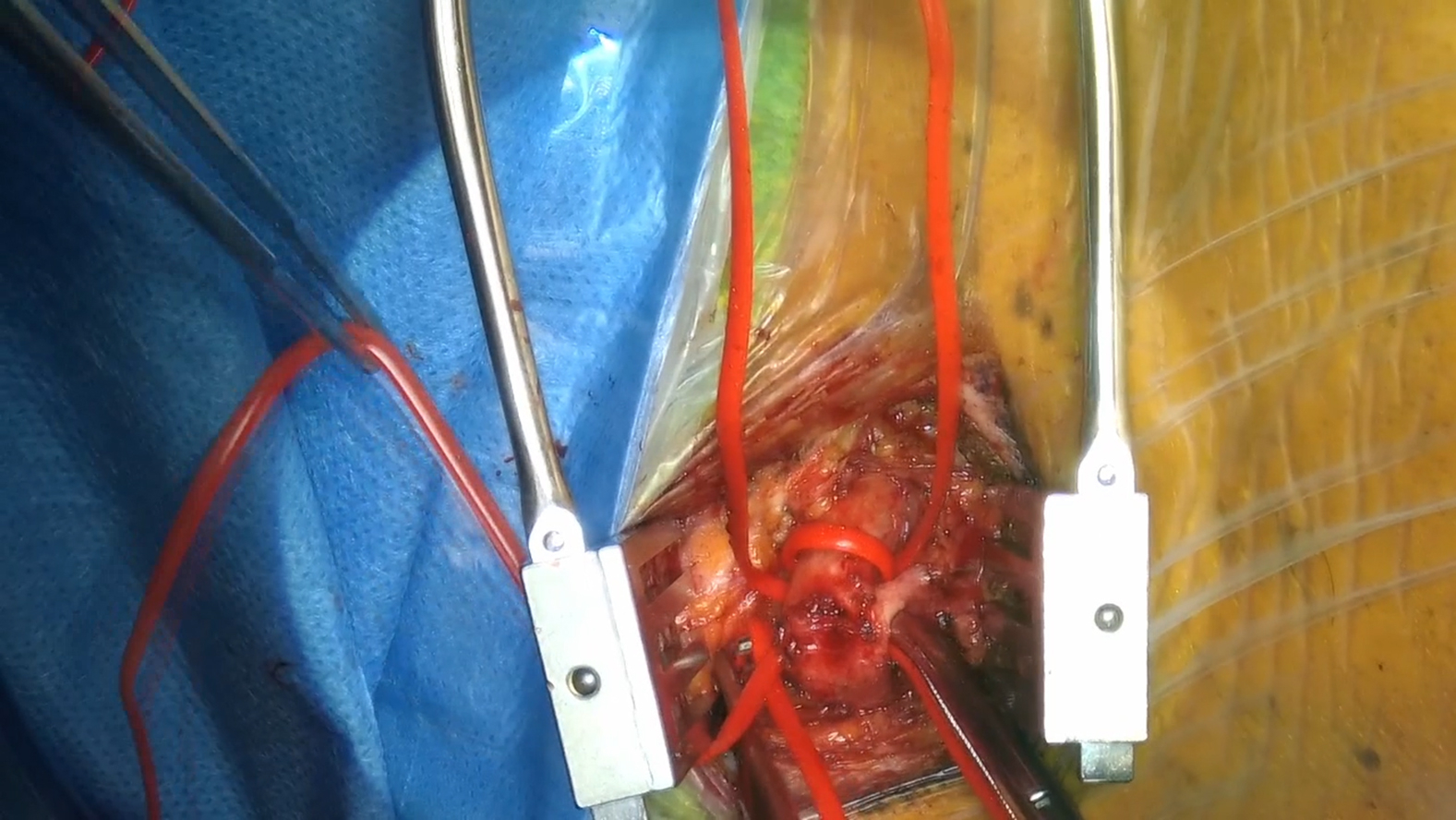

Supplement: Video 1 — Redo right axillary artery cannulation using direct cannulation technique in a patient with prior direct axillary cannulation. Video available at: https://www.jtcvs.org/article/S2666-2507(25)00237-8/fulltext. [file fx2.jpg]
